# Contrasting Methane, Sulfide and Nitrogen‐Loading Regimes in Bioreactors Shape Microbial Communities Originating From Methane‐Rich Coastal Sediment of the Stockholm Archipelago

**DOI:** 10.1111/1462-2920.70056

**Published:** 2025-02-16

**Authors:** Maider J. Echeveste Medrano, Garrett J. Smith, Irene Sánchez‐Andrea, Mike S. M. Jetten, Cornelia U. Welte

**Affiliations:** ^1^ Department of Microbiology Radboud Institute for Biological and Environmental Sciences, Radboud University Nijmegen the Netherlands; ^2^ Department of Microbiology Ohio State University Columbus USA; ^3^ Center of Microbiome Science The Ohio State University Columbus USA; ^4^ Department of Environmental Sciences for Sustainability IE University Segovia Spain; ^5^ Laboratory of Microbiology Wageningen University and Research Wageningen the Netherlands

**Keywords:** Aquatic microbiology, Climate change microorganisms, Element cycles and biogeochemical processes, Metabolism, Metagenomics/community genomics, Microbial communities, Uncultured microbes

## Abstract

Coastal ecosystems are increasingly exposed to high nutrient loads and salinity intrusions due to rising seawater levels. Microbial communities, key drivers of elemental cycles in these ecosystems, consequently, experience fluctuations. This study investigates how the methane‐rich coastal sediment microbiome from the Stockholm Archipelago copes with high and low nitrogen and sulfide loading by simulating coastal conditions in two methane‐saturated anoxic brackish bioreactors. Over a year, the bioreactors were subjected to the same ratio of nitrate, ammonium and sulfide (2:1:1) under eutrophic or oligotrophic conditions and monitored using 16S rRNA gene amplicon and metagenomic sequencing. Sulfide was depleted in both conditions. Sulfide‐dependent denitrification was the predominant process in eutrophic conditions, whereas dissimilatory nitrate reduction to ammonium dominated under oligotrophic conditions. Methane oxidation was driven by *Methylobacter* and *Methylomonas* in eutrophic conditions, whereas a more diverse methane‐oxidising microbial community developed under oligotrophic conditions, which likely competed for nitrate with anaerobic methanotrophic archaea and the gammaproteobacterial MBAE14. Novel putative copper‐dependent membrane‐bound monooxygenases (Cu‐MMOs) were identified in MBAE14 and co‐enriched *Rugosibacter* genomes, suggesting the need for further physiological and genetic characterisation. This study highlights the importance of understanding coastal anoxic microbiomes under fluctuating conditions, revealing complex interactions and novel pathways crucial for ecosystem functioning.

## Graphical abstract

In this study, microbial communities are enriched in brackish anoxic bioreactors with methane, sulfide, nitrate and ammonium. Novel diversity is identified and functionally analysed.

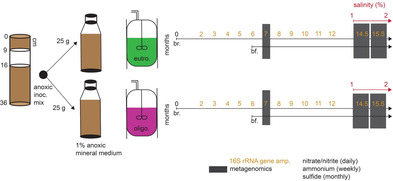

## 1 Introduction

By bridging freshwater and seawater bodies, coastal ecosystems are kinetic drivers of microbial carbon, nitrogen, and sulfur cycling (Siefert and Plattner 2004). Methane is becoming a greenhouse gas of increasing concern in these anthropogenically and climate change‐exposed environments (Howarth et al. 2011; Rosentreter et al. 2021; Saunois et al. 2016, 2020). Methane is produced by methanogenic archaea using a limited number of substrates, whereas the consumption of methane in the sediment is mostly attributed to consortia of sulfate‐reducing bacteria and anaerobic methane‐oxidising archaea, or to aerobic methane‐oxidising bacteria (MOB) in the oxygenated sediment layers or water column (Kalyuzhnaya, Gomez, and Murrell 2019; Venetz et al. 2023; Wallenius et al. 2021; Welte et al. 2016). There is growing evidence that oxidised nitrogen compounds and metalloids could be used as electron acceptors by methanotrophs (Glodowska, Welte, and Kurth 2022; He et al. 2019). Still, methane oxidisers share the same niche and compete for electron acceptors in coastal sediments with other microbial guilds such as nitrifiers (oxygen), anaerobic ammonium oxidisers (nitrite) and denitrifiers (NOx) or sulfide oxidisers (oxygen, NOx) (Kuypers, Marchant, and Kartal 2018; Wu et al. 2021). We currently lack detailed knowledge on how coastal microbial communities could be affected by long‐term nutrient loading or salinity increase. Likewise, we do not yet know how the methane‐oxidising communities cope with or adapt to such challenges.

Coastal microbiomes, including the methane oxidisers, are subjected to various stressors, such as deoxygenation leading to high ebullitive methane fluxes (Żygadłowska et al. 2024), eutrophication due to agricultural runoff and human sewage release (Tuholske et al. 2021), sulfide toxicity that is compromising the microbial methane filter (Dalcin Martins et al. 2024) and salt intrusion leading to freshwater salinisation syndrome (Kaushal et al. 2021). Fortune et al. investigated eutrophication‐derived shifts in key nitrogen functional marker genes in tidal systems and observed a negative correlation between the nitrous oxide reduction gene *nosZ* involved in denitrification and excess nutrient load, suggesting that microbial marker genes could be used as a monitoring tool for the trophic status of the ecosystem (Fortune et al. 2024). Laboratory‐scale bioreactor cultivations enable us to observe the results of specific stressors on the microbial community composition without the confounding factors of a highly dynamic natural ecosystem, leading to the discovery of novel taxa potentially involved in driving nutrient cycling under eutrophic conditions in the environment (Arshad et al. 2017; Dalcin Martins et al. 2022; Delgado Vela et al. 2021) or observing the effect of salinisation on ammonium and methane‐oxidising microbial communities in a controlled environment (Frank et al. 2023). Moreover, there is an untapped potential for novel metabolism associated with chemolithotrophic coastal microbiomes as many microbial taxa of such microbiomes cannot be assigned to known references (Dalcin Martins et al. 2024).

In this study, we investigated the effects of high and low nitrogen (nitrate and ammonium) and sulfide loading under high methane concentrations on the microbial community structure and function of a coastal anoxic sediment microbiome. Moreover, to assess the effect of salt intrusion on nutrient cycling, we analysed the effect of salinity increase on a long‐term brackish‐acclimated culture. We conducted a long‐term continuous bioreactor study by monitoring physicochemical parameters, 16S rRNA gene amplicon and metagenomic sequencing of the two differing bioreactors with a focus on the methanotrophic and sulfide‐oxidising community performing denitrification or dissimilatory nitrate reduction (DNRA). In the process, we co‐enriched a member of the betaproteobacterial MBAE14‐like family (*Pseudomonadales* IMCC2047) and betaproteobacterial *Rugosibacter* species (order *Nitrosomonadales*) with genomes containing putative novel copper‐dependent membrane‐bound monooxygenases (Cu‐MMOs).

## 2 Experimental Procedures

### 2.1 Inoculum and Bioreactors Operation

Two 6.8 L bioreactors (Applikon, Delft, The Netherlands) were operated as sequencing batch reactors (SBRs) for 15 months (schematic overview of experimental procedures in Figure S1). From months 3–4 onwards, a wall‐associated biofilm developed (Figure S2). Both bioreactors were inoculated anoxically with sediment: each received 25 g from a mixture of all samples of depths 9–16 cm from the 2019 Stockholm Archipelago's coastal anoxic sediments campaign (Site 3, Sandöfjärden) (Dalcin Martins et al. 2024), where a high methanotrophic activity was observed. Biomass was stored in fully anoxic conditions at 4°C in sealed aluminium bags (Gruber‐Folien GmbH & Co. KG, Germany) until April 4th, 2021. On that date, the samples were mixed with a mineral medium containing (per 1 L): 1.25 mL of CaCl₂·2H₂O (192 mg/L), 0.6 mL of KH₂PO₄ (100 g/L), 1.25 mL of MgSO₄·7H₂O (288 mg/L), 0.5 mL of DAMO trace elements, 0.6 mL of anammox trace elements and 0.6 mL of FeSO₄, as specified in Arshad et al. (2017). The mixture was prepared under brackish salinity conditions (1% Red Sea, Red Sea, Israel) and subsequently used for bioreactor inoculation. Nitrate, ammonium and sulfide loading were slowly built up using a 2:1:1 molar ratio, starting with lower concentrations to adapt the microbial community to the nutrient concentrations and ensure complete sulfide conversion (Figure S3). By the end of the 10‐month monitoring, the eutrophic bioreactor was in a steady state and received around 8 mmol nitrate, 4 mmol ammonium and 4 mmol sulfide, while the oligotrophic bioreactor was supplied with approximately 0.4 mmol nitrate, 0.2 mmol ammonium and 0.2 mmol sulfide (Figures S1 and S3). The sulfide source employed was sodium sulfide hydrate 60%–64% (Na_2_S x 3H_2_O) (Acros Organics, Thermo Fischer Scientific, The Hague, The Netherlands); an anoxic stock solution was prepared that was refreshed approximately every 10 days. Nitrate and ammonium were supplemented in the form of NaNO_3_ (VWR, Radnor, Pennsylvania, USA) and NH_4_Cl (Fischer Scientific, Gell, Belgium). Medium supply (1 L) and sulfide (0.2 L dissolved in mineral medium) were always provided from separate bottles. The SBR cycle consisted of 23 h of medium influent inflow, 10 min of settling and 50 min of reactor liquid removal. The hydraulic retention time was approximately 4 days (1.2 L/day reactor liquid removal). Both bioreactors were operated at 150 rpm with stirrers that contained one standard six‐blade and a double‐blade spiral turbine. The reactors were saturated with methane by receiving CH_4_:CO_2_ (95:5) at 10 mL/min. The bioreactors were flushed with Ar/CO_2_ (95:5) inflow during reactor settling to ensure anoxic conditions at all times. The pH of the bioreactor was monitored with a pH electrode (Applisense, Applikon, Delft, The Netherlands), maintained at pH 7 with 1 M KHCO_3_ solution controlled with a pH pump by an ADI 1010 biocontroller (Applikon, Delft, The Netherlands). To subject the microbiomes to a second environmentally relevant coastal microbiome stressor, the salinity was increased to 2% Red Sea Salt in the last month of operation (13.5–14.5 months) (Figure S1).

### 2.2 Physicochemical Measurements

The bioreactor was checked daily for nitrate and nitrite concentrations with MQuantTM colorimetric test strips (Merck, Darmstadt, Germany). Monthly sulfide was either measured in liquid samples with the methylene blue assay (Moest 1975) (HACH, Loveland, CO, USA) or in the headspace as gas using a 7890B GC System (Agilent Technologies, Santa Clara, CA, USA) (Figure S3). Liquid samples were collected every week for ammonium measurements (Figures S1 and S3). Ammonium was determined fluorescently using a high sensitivity protocol (range from 40 to 400 μM): 2‐mercaptoethanol created reduced conditions throughout the entire mixture, allowing it to react with ortho‐phthaldialdehyde (Taylor et al. 1974). Fluorescence was recorded via a Spark 10 M Plate Reader (Tecan, Grodig, Austria).

### 2.3 Nucleic Acid Extraction

DNA samples for downstream 16S rRNA gene amplicon sequencing were obtained in months: 0 (inoculation day), 2, 3, 5–12, 14.5 (before salt increase to 2% NaCl) and a month afterwards, 15.5 (Figure S1). A total volume of 40 mL of biomass was extracted from both bioreactors. Samples were then centrifuged for 5 min at maximum speed, and the remaining pellet was used for DNA extraction. From month 6 onwards, DNA was also extracted from the wall‐associated biofilm by using a sterile Sigma SIAL0010 cell scraper (Sigma Aldrich, MO, USA) (Figures S1 and S2). The biofilms covered both bioreactors' heights between 3.8 L and 5 L, corresponding to the minimum and maximum volumes of the SBR cycle (Figure S2). DNA extraction for metagenomic sequencing was performed in triplicates in bioreactors, planktonic and wall biofilm samples, on months: 7, 14.5 and 15.5.

DNA extractions were performed using the Power Soil Kit (Qiagen, Hilden, Germany), with a modified initial bead‐beating step with solution C1 at 10 min 50 oscillations per second and PowerBead tubes on a TissueLyser LT (Qiagen, Hilden, Germany). DNA quantities were determined by Qubit using the dsDNA HS Kit (Thermo Fisher Scientific, Waltham, MA, USA). DNA quality was determined using the NanoDrop Spectrophotometer ND‐1000 (Isogen Life Science, Utrecht, The Netherlands). Our analysis included 16S rRNA gene amplicon and metagenomics raw sequencing data obtained from Dalcin Martins et al. Stockholm Archipelago sediment DNA extractions, under NCBI BioProject PRJNA805085. Raw sequencing data originated from Site 3 (Sandöfjärden) Stockholm Archipelago sediment campaign 2019 and included 16S rRNA gene amplicon depths (in cm) 9,10,12,14,16‐ and metagenomic depth (in cm): 0–4, 9–12, 21–24 and 33–36 analysis (Dalcin Martins et al. 2024).

### 2.4 16S rRNA Gene Amplicon Analysis

Gene amplicon sequencing was performed by Macrogen Europe BV (Amsterdam, The Netherlands) on a MiSeq Illumina platform, using library kit Herculase II Fusion DNA Polymerase NEXTERA XT Index kit V2 (Illumina, Eindhoven, Netherlands), generating 2×300bp paired‐end reads. Bacterial primers were Bac341F (CCTACGGGNGGCWGCAG) (Herlemann et al. 2011) and Bac806R (GGACTACHVGGGTWTCTAAT) (Caporaso et al. 2012), whereas archaeal primers were Arch349F (GYGCASCAGKCGMGAAW) and Arch806R (GGACTACVSGGGTATCTAAT) (Takai and Horikoshi 2000).

Obtained 16S rRNA gene amplicon raw sequences were processed in R Studio version v1.2.5033 and R v4.0.4 with: DADA2 package v1.18, ggplot2 v3.3.5, phyloseq v1.32, vegan v2.5.6, DESEQ2 1.28.1, dendextend v1.14, tidyr v1.1.3, viridis v0.5.1, reshape v0.8.8, zoo v1.8.8 and plyr v1.8.6. Briefly, we used the DADA2 pipeline (Callahan et al. 2016) quality plots to trim forward and reverse bacterial reads at 295 bp and 260 bp while archaeal reads at 295 bp and 220 bp, respectively. Trimming was combined with the trimLeft option 20 for primer removal. After error models were generated, sequences were dereplicated and merged, and chimeras were discarded, producing between 42.000 and 88.000 paired‐end non‐chimeric merged bacterial or archaeal sequences. Later, amplicon sequencing variants (ASVs) were inferred and assigned using the Silva database release v.138.1 (Quast et al. 2013) downloaded from https://zenodo.org/record/4587955#YdgiLBPMI‐R. ASVs were clustered by taxonomy and relative abundance using the R package phyloseq (McMurdie and Holmes 2013) and plotted with ggplot2.

### 2.5 Metagenomics Analysis

Samples were sequenced by Macrogen Europe VB (Amsterdam, The Netherlands) with a TruSeq DNA PCR free library using an insert size of 350 bp on a NovaSeq6000 Illumina platform, producing 2×151bp paired‐end read (10Gbp/sample). Metagenomic data were analysed as follows. Read quality was assessed with FASTQC v0.11.8 before and after quality trimming, adapter removal and contaminant filtering, performed with BBDuk (BBTools v38.75). Trimmed reads were co‐assembled *de novo* using metaSPAdes v3.14.1 (Nurk et al. 2017) and mapped to assembled contigs using BBMap (BBTools v38.75) (Bushnell 2022). Sequence mapping files were handled and converted using SAMtools v1.10. Contigs at least 1000‐bp long were used for binning with CONCOCT v1.1.0 (Alneberg et al. 2014), MaxBin2 v2.2.7 (Wu, Simmons, and Singer 2016) and MetaBAT2 v2.1512 (Kang et al. 2019). Resulting metagenome‐assembled genomes (MAGs) were dereplicated with DAS Tool v1.1.213 (Sieber et al. 2018) and taxonomically classified with the Genome Taxonomy Database Toolkit GTDB‐Tk v1.3.0 (Chaumeil et al. 2019) release 9514. MAG completeness and contamination were estimated with CheckM v1.1.2 (Parks et al. 2015). MAGs were renamed to their lowest GTDB‐Tk category. MAGs were annotated with DRAM v1.0 (Shaffer et al. 2020) with default options, except min_contig_size at 1000 bp, and genes of interest were searched in annotation files as well as via BLASTP and HMM analyses. Gene‐based metagenomic analysis was resolved using CoverM v0.6.1 (https://github.com/wwood/CoverM) with contig flag, minimum of 95% identity and aligned read length of 75% for each read. Here, KEGG‐curated HMM profiles of selected functional marker genes were mapped against our metagenome and corrected for read and contig size. The motivation to employ certain genes for sulfide detoxification/oxidation (sulfide: quinone oxidoreductase, *sqr*), DNRA (nitrite: ammonium oxidoreductase, *nrfA*) and denitrification (nitrate reductase [*narG*, *napA*], nitrite reductase [*nirS*, *nirK*], nitric oxide reductase [*norB*] and nitrous oxide reductase [*nosZ*]) was based on pathway specificity, well‐curated genes (KEGG‐based) with known directionality, except for nitrate reductase/nitrite oxidoreductase (*narG/nxrA*), which was put in context with downstream denitrification genes.

Genomes containing Cu‐MMO were downloaded from JGI's IMG/MER after identification of Pfam02461 using the pfam search of their ‘all isolate’ database, noting that several genomes in the database appear to be MAGs. Several additional genomes were downloaded due to shared taxonomy or identification of the pfam motif in other automated mining efforts of large genomic databases (e.g., AnnoTree, Mendler et al. 2019). Reference genomes were annotated using DRAM (v1.2.2) (Shaffer et al. 2020) to homogenously call genes and identify Cu‐MMOs. The amino acids of the A subunits of Cu‐MMOs were aligned using MAFFT's ‘ginsi’ setting (1000 iterations, global alignment; v7.397) (Katoh and Standley 2013), trimmed automatically using Trimal (v1.4.rev22) with default settings (Capella‐Gutiérrez, Silla‐Martínez, and Gabaldón 2009) and the alignment input into IQ‐Tree (1.6.12) with 1000 bootstraps and automatic model selection (Nguyen et al. 2015). Ammonia‐oxidising bacteria references employed to analyse the operon architecture of *amoCABEDcycAB* and *haoAB* included: *Nitrosococcus oceanii* strain ATCC 19707, ‘*Ca*. Nitrosacidococcus tergens’ sp. RJ19 (Picone et al. 2021) and *Nitrosomonas europaea* strain ATCC 19718. For the pyrroloquinoline quinone (PQQ)‐ADH phylogenetic tree generation, reference (Keltjens et al. 2014) and study sequences were first aligned using with default MAFFT setting from EMBL‐EBI web browser (Madeira et al. 2022), trimmed with (−gappy out) using Trimal v1.4.rev15 (Capella‐Gutiérrez, Silla‐Martínez, and Gabaldón 2009) and ran with IQ‐TREE v2.0.3 using flags‐st AA‐m MFP‐bb 1000‐nt AUTO (Nguyen et al. 2015). Fast amino acid identity (AAI) matrixes were generated with the online tool of the Kostas lab with default settings (Rodriguez‐R and Konstantinidis 2016). The biogeography of ‘*Ca*. Methanoperedens BLZ2’ and the *Methylomonadaceae* family was conducted using SingleM‐resolved Sandpiper (April 2024) (https://sandpiper.qut.edu.au/).

## 3 Results

### 3.1 Fluctuations in Sulfide, Nitrate, Nitrite and Ammonium Concentrations in Between Substrate Loadings

**FIGURE 1:**
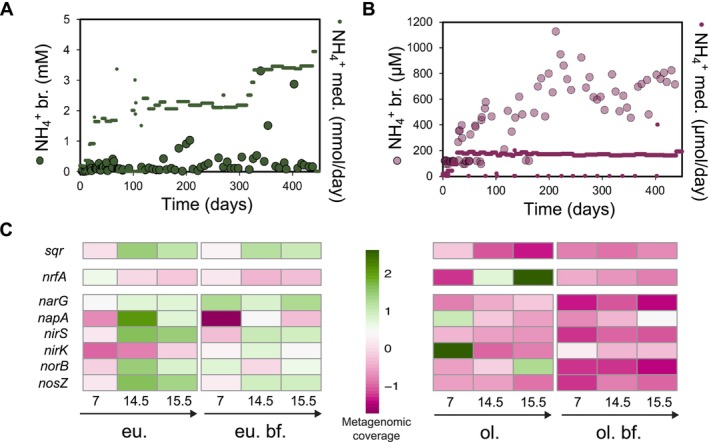
(A, B) Ammonium added to the bioreactor (in mmol/day or in μmol/day) and measured in situ in the bioreactor (br) (in mM or μM) weekly over the course of the experiment in the eutrophic (A) and oligotrophic system (B). Both primary and secondary y‐axis indicate the same scale but with different units. (C) Z‐score normalised gene coverage of selected functional markers for sulfide detoxification (*sqr*), Dissimilatory Nitrate Reduction to Ammonium (DNRA, *nfrA*) and denitrification (*narG, napA, nirS, nirK, norB, nosZ*). Note that the high and low colouring scheme sustains the green (high) and low (pink) pattern for all panels.

For the eutrophic bioreactor, sulfide was fully consumed during the monitoring period, whereas ammonium remained below 0.5 mM, and nitrate fluctuated between 2 and 7 mM (Figure S3). Nitrite did not accumulate to more than 1–5 μM except after substrate loading when mM nitrite levels were reached for a short time (Figure 1A and Figure S3). In the eutrophic bioreactor, nitrite accumulated at 80–100 days to about 8 mM, resulting in a temporary stop of nitrate and sulfide addition until all nitrite was converted. From months 3–4 onwards, a biofilm developed on the wall of the eutrophic bioreactor that was separately sampled and sequenced from month 6 onwards (Figures S1 and S2). The oligotrophic bioreactor received 400 μM nitrate, 200 μM ammonium and 200 μM sulfide per day. Here, sulfide was also fully consumed whereas ammonium remained below 200 μM and nitrate was around 200 μM (Figure S3). With some exceptions, nitrite did not accumulate (Figure 1B and Figure S3). Ammonium showed an increasing trend after 100 days, indicating a permanent shift from denitrification to DNRA (Figure 1B). The oligotrophic bioreactor biofilm developed earlier, at months 1–2. The oligotrophic system had a denser and more uniform biofilm compared to the eutrophic bioreactor (Figure S2).

### 3.2 Oligotrophic Microbiome Sustained DNRA While Denitrification Dominated Eutrophic System

Sulfide was fully depleted in both bioreactors, suggesting an active (nitrate‐dependent) sulfide‐oxidising microbial community (Figure S3). Denitrification dominated nitrate conversion in the eutrophic system as only a minor part was recovered as ammonium (ranging between 0.5 mM and 1 mM) (Figure 1A). In contrast, the oligotrophic bioreactor produced increasing amounts of ammonium over time (Figure 1B): the concentration rose from 200 μM to around 800 μM, indicating that DNRA was more important than denitrification as the nitrate reduction route.

To underpin the processes for sulfide conversion and nitrate removal, we investigated the metagenomic read coverage of functional marker genes for these reactions: *sqr* for sulfide oxidation/detoxification, *nrfA* for DNRA and six genes (*narG, napA, nirS, nirK, norB, nosZ*) for denitrification. Coinciding with the higher loading of sulfide (Figure S3), the coverage of *sqr* appeared to be higher in the eutrophic system (Figure 1C). In agreement with the observed ammonium production in the oligotrophic bioreactor, we found increased metabolic potential for DNRA in this system through a particularly high mean coverage of *nrfA* in the middle (month 7) and end (months 14.5–15.5) of the monitoring, respectively (Figure 1C). Overall, denitrification marker genes showed a higher coverage in the eutrophic system compared to the oligotrophic system (Figure 1C).

### 3.3 Substrate Loading Affects Microbial Community Composition Dominated by Gammaproteobacterial Groups

**FIGURE 2:**
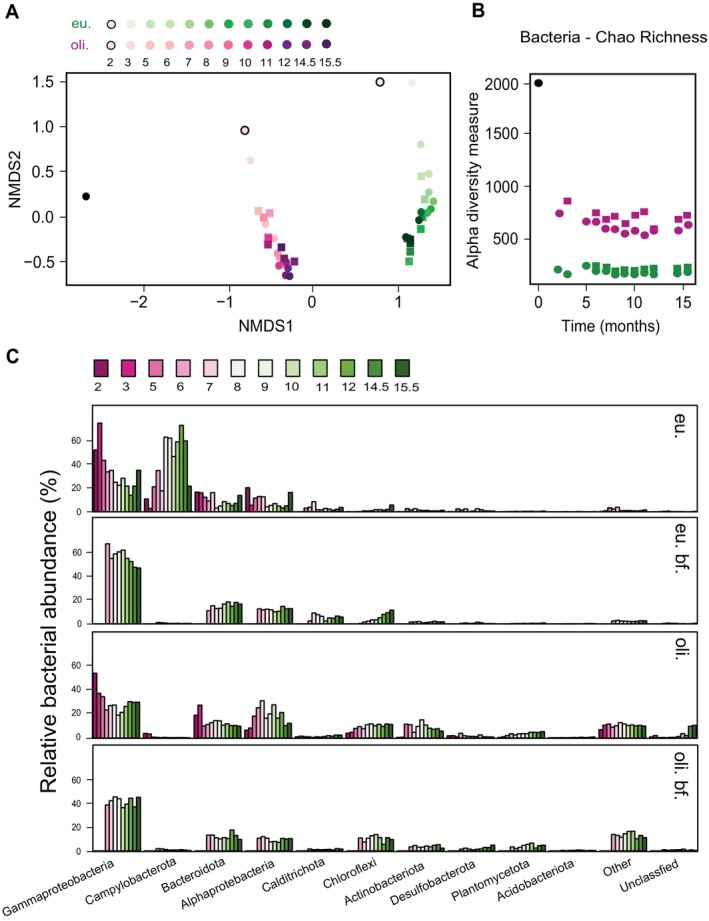
(A) Nonmetric multidimensional scaling (NMDS) based on Bray–Curtis dissimilarity of 16S rRNA gene bacterial amplicon sequencing variants (ASVs) per bioreactor substrate loading. Dimensionality was set at *k* = 3 and recorded a stress value of 0.04571603. Filled circle, inoculum; black outlined circle, month 2 sample; circles, planktonic samples; squares, biofilm samples; pink symbols, oligotrophic bioreactor; green symbols, eutrophic bioreactor. (B) Bacterial 16S rRNA gene ASVs richness index was calculated via Chao index and indicated as Alpha diversity measure (y‐axis) per sample source across time (x‐axis). Black circle, inoculum; circles, planktonic samples; squares, biofilm samples; pink symbols, oligotrophic bioreactor; green symbols, eutrophic bioreactor. (C) From top to bottom top 5% bacterial 16S rRNA gene ASVs reads recovered at phylum level for all time points and substrate loading conditions. Samples were ordered from eutrophic (eu) to oligotrophic (oli.), including biofilm (bf) from month 6 onwards.

First, we verified that the microbial community of the inoculum was representative of the original mixture from depths of 9–16 cm (referred to as ‘raw’) (see Tables S1 and S2). By month 2, the bacterial community showed a marked difference compared to the inoculum in both systems, accompanied by a strong decrease in richness and diversity (Figure 2A,B, Figure S4). The diversity decrease took longer in the archaeal community (Figure S4). The salinity increase (from 1% to 2%) in the last month of operation did not influence the brackish‐acclimated microbiomes performance (Figure 2A,B and Figure S4).

We recovered a total of 452 bacterial and 17 archaeal MAGs from the combined datasets (Table S3). From these 469 MAGs, only 79 were highly curated bacterial and one archaeal (> 90% complete and < 5% contaminated) with > 1% of mapped reads for any bioreactor time point, excluding the inoculum sediment depths (Figure S5). The single archaeal genome belonged to nitrate‐dependent anaerobic methanotroph ‘*Ca*. Methanoperedens BLZ2’ sp. (Figure S5). The Gammaproteobacterial MAGs dominated both bioreactor systems, with 22/79 of the dominating and curated MAGs belonging to the group (Figure S5). Moreover, the gammaproteobacteria showed a high bacterial 16S rRNA gene amplicon read abundance (40%–70%), while they were not so abundant in the inoculum (~10%) (Figure 2C and Figure S6). In the eutrophic system, Campylobacterota increased in abundance over time in the planktonic biomass. A total of 70 ASVs were identified, representing five different species (counts in parenthesis): *Sulfurimonas* (39), *Sulfurovum* (16), *Sulfuricurvum* (3), *Sulfurospirillum* (2), *Pseudarcobacter* (5) and unknown (5). The overall diversity in the eutrophic biofilm did not change much during the reactor operation (Figure 2C). In both the planktonic and biofilm biomass of the oligotrophic system, the major phyla remained evenly represented after 2 months of operation. There was a higher abundance of ‘other’ phyla compared to the eutrophic system (Figure 2C and Figure S6).

### 3.4 Distinct Substrate Loading Defines Methanotrophic Community Structures With Repercussions on the Nitrogen Cycle

**FIGURE 3:**
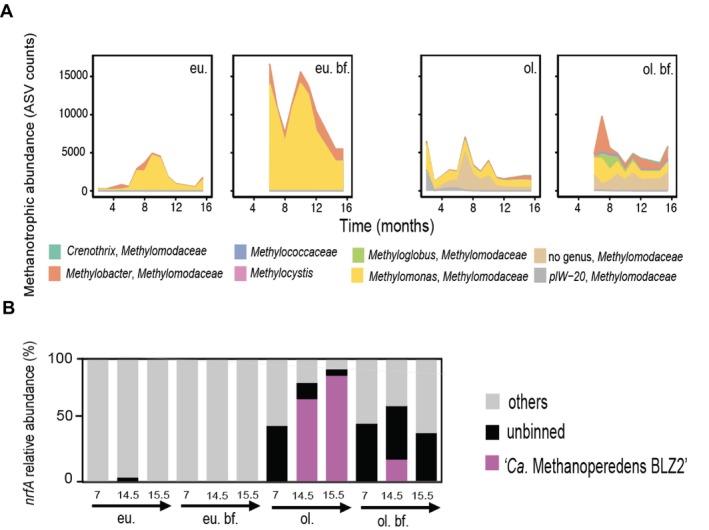
(A) Total bacterial amplicon sequencing variants (ASVs) recovered for all methanotrophic families across time and conditions: Eu(trophic) or, ol(igotrophic) and with/without biofilm (bf.). (B) Percentage of top (> 10%) methanotrophic genome contribution to functional marker gene coverage for dissimilatory nitrate reduction to ammonium (DNRA): *NrfA*.

We proceeded to disentangle the abundance and contributions of the methanotrophic populations in these communities. Despite the absence of oxygen via air supply and high amounts of reducing sulfide and nitrate, a variety of MOBs were enriched in both systems. Type I MOB belonging to *Methylomonadaceae* (gammaproteobacteria) showed the highest abundance in nearly all systems and niches (Figure 3A and Figure S5). In the eutrophic system, we retrieved between 0.5% (bioreactor month 3) to as much as 38.6% (biofilm month 10) bacterial ASV reads of this taxon (Figure 3A and Table S1). In the oligotrophic system, we recovered between 1.5% (bioreactor month 3) and 16.8% (biofilm month 7) of *Methylomonadaceae* ASV reads (Figure 3B and Table S1). The oligotrophic system appeared to have a slightly less abundant (ASV counts), yet more diverse, community of methanotrophs compared to the eutrophic one, including more representatives of *Crenothrix*, *Methylobacter* or *Methylomonadaceae* methanotrophs for which no genus could be assigned based on 16S rRNA gene sequence (Figure 3A). For both the eutrophic and oligotrophic systems, the MOB community was more abundant in the biofilm than in the bioreactor (double the amount of ASV counts) (Figure 3A, Figure S5 and Tables S1. and S3), whereas the anaerobic methanotrophic archaeon ‘*Ca*. Methanoperedens’ thrived better in the oligotrophic bioreactor, being present both in the bioreactor suspension and the biofilm (Table S2, Figure S7).

In the oligotrophic system, ‘*Ca*. Methanoperedens BLZ2’ (with different 16S rRNA gene ASVs identified) contributed to more than 89.2% of the total archaeal ASV reads recovered from month 7 onwards and sustained its presence over time, with 99.4% of the archaeal 16S rRNA gene amplicon reads in month 15.5 in the bioreactor (matching as much as 21% of the total metagenomic reads for the same month and system) (Figure S5 and Table S2). On the contrary, the eutrophic system showed only traces of ‘*Ca*. Methanoperedens’ from month 11 onwards (excluding the biofilm) despite being a system saturated in methane and excess nitrate (Figures S5 and S7 and Table S2).

**FIGURE 4:**
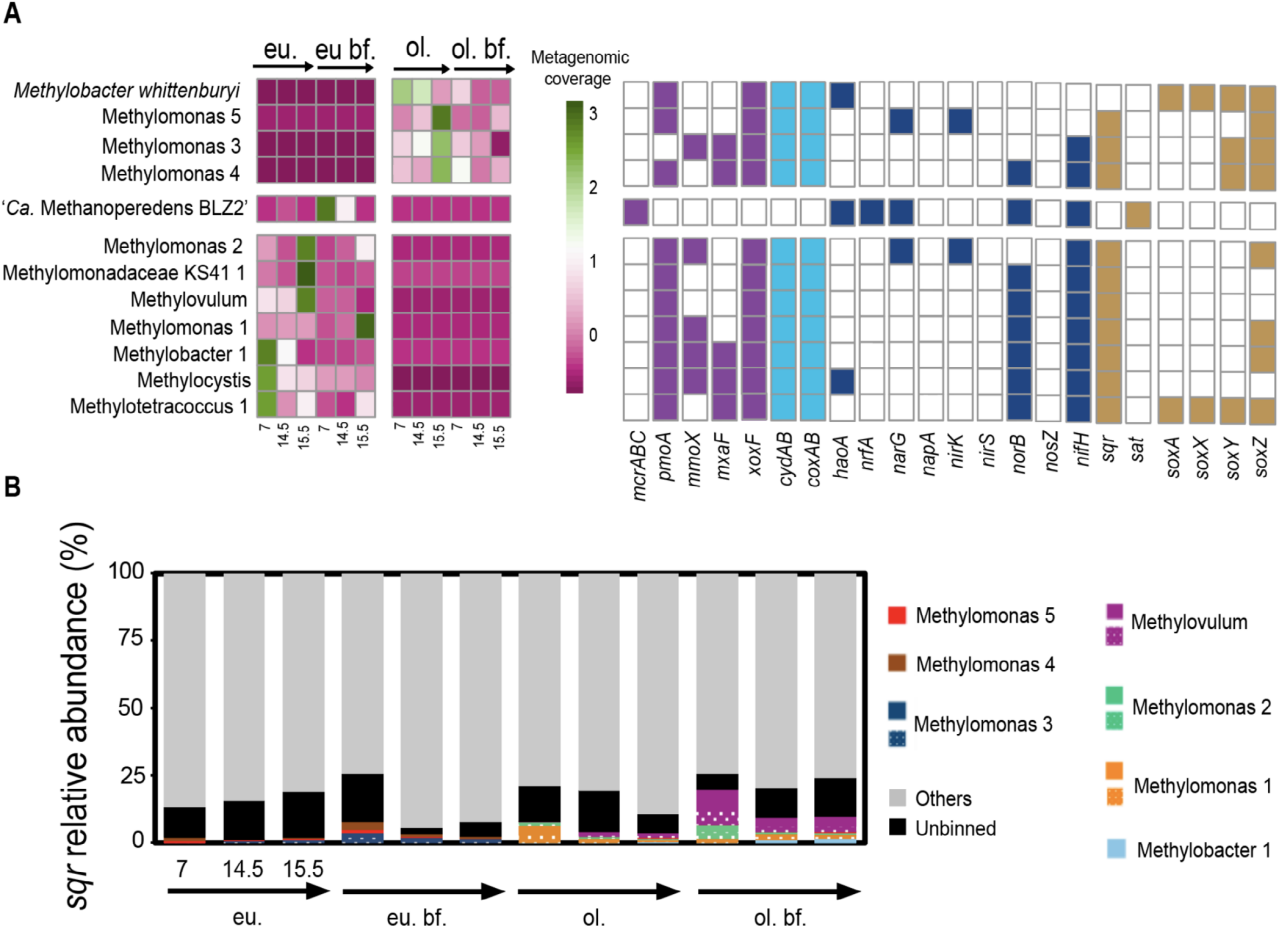
(A) Functional annotation of high‐quality (> 86% complete and < 8% contaminated) metagenome‐assembled genomes (MAGs) of methanotrophs in two bioreactor systems. MAGs were clustered based on Z‐score mean normalised coverage patterns depicted using a heatmap with individual rows depicting the time of sampling (7, 14.5, 15.5 months) and location in bioreactor sample (eu., eutrophic; eu.bf., biofilm eutrophic; ol., oligotrophic; ol.bf., oligotrophic biofilm). Functional marker genes are depicted as presence (filled)/absence (empty) matrix with colour coding for metabolism. Purple, methane metabolism; light blue, oxidases; dark blue, nitrogen metabolism; orange, sulfur metabolism. Marker genes encoding for (part) of the following enzymes were queried against the MAGs: *McrABC*, methyl coenzyme M reductase; *pmoA*, particulate methane monooxygenase; *mmoX*, soluble methane monooxygenase; *mxaF*, Ca‐dependent methanol dehydrogenase; *xoxF*, La‐dependent methanol dehydrogenase; *cydAB*, cytochrome *bd* oxidase; *coxAB*, cytochrome *c* oxidase; *haoA*, hydroxylamine dehydrogenase; *nrfA*, nitrite: Ammonium oxidoreductase; *narG*, nitrate reductase; *napA*, nitrate reductase (periplasmic); *nirK/nirS*, nitrite reductase; *norB*, nitric oxide reductase; *nosZ*, nitrous oxide reductase; *nifH*, nitrogenase; *sqr*, sulfide:Quinone oxidoreductase; *sat*, sulfate adenylyl transferase; *soxAXYZ*, sulfur oxidation system. (B) Percentage methanotrophic MAG contribution to the sulfide quinone oxidoreductase (*sqr*) gene coverage (> 1%) during the three sampled months (7, 14.5, 15.5) and in the different bioreactor samples (eu., eutrophic; eu.bf., biofilm eutrophic; ol., oligotrophic; ol.bf., oligotrophic biofilm). Colour coding indicates which MAGs contributed more to the sample's *sqr* gene coverage. MAGs that harboured two gene copies were indicated with and without dot filling. MAGs were classified to the lowest known GTBD‐Tk taxonomical category. Others category (grey) refer to the rest of non‐methanotrophic MAGs.

The methane‐oxidising microbial community was intricately linked with the nitrogen cycling in the bioreactors. It seemed that DNRA observed in the oligotrophic bioreactor was primarily performed by ‘*Ca*. Methanoperedens BLZ2’ sp. (Figure 3B) whereas the assimilatory *nirB* was more widely distributed among different MOB (Figure S8). Although denitrification potential was encoded in several *Methylomonas* genomes in the eutrophic system (Figure 4A, Figure S8 and Table S4), it was clear that non‐methanotrophic MAGs had a much larger contribution to denitrification, largely sulfide oxidisers including *Thiobacillus* and *Sulfurimonas* in the eutrophic bioreactor and *Magnetovibrio*, *Thiomonas* and *Thiothrix* in the oligotrophic reactor (Figure S8, Tables S1 and S6).

### 3.5 Oligotrophic Bioreactor's Microaerophilic and Sulfide‐Tolerant Methanotrophic Community Members Were Metabolically More Diverse and Unexplored

To get a better understanding of C, N and S cycling potential of the methanotrophic members of the communities, we investigated 12 high‐quality MOB MAGs and then clustered them based on abundance patterns (Figure 4A, Figure S5 and Table S4). More than half of these MAGs belonged to the *Methylomonadaceae* family, with *Methylomonas*, *Methylovulum*, *Methylobacter* and subgroup *Methylomonadaceae* KS41 as genera. While the MAGs *Methylovulum* and *Methylomonadaceae* subgroup KS41 were more abundant in the oligotrophic system, *Methylomonas* and *Methylobacter* got more enriched in the eutrophic system (Figure 4A and Figure S5). Based on average AAI, all *Methylomonadacae* MAGs, except for the ones labelled ‘*Methylomonas whittenburyi*’ and Methylomonas 4, may represent new species (Figures S9 and S10).

All MOB MAGs encoded for soluble or particulate methane monooxygenase (sMMO or pMMO) together with *xoxF*‐encoded lanthanide‐dependent methanol dehydrogenases (MDHs, Figure 4A). Some MAGs also encoded the calcium‐dependent MDHs (Figure 4A). Many of the MOB MAGs seemed capable of detoxifying sulfide via *sqr* or included *sox* genes (Figure 4A,B).

### 3.6 Enrichments of MBAE14‐Like Pseudomonadales IMCC2047 and *Rugosibacter* With Atypical Cu‐MMO Genes

**FIGURE 5:**
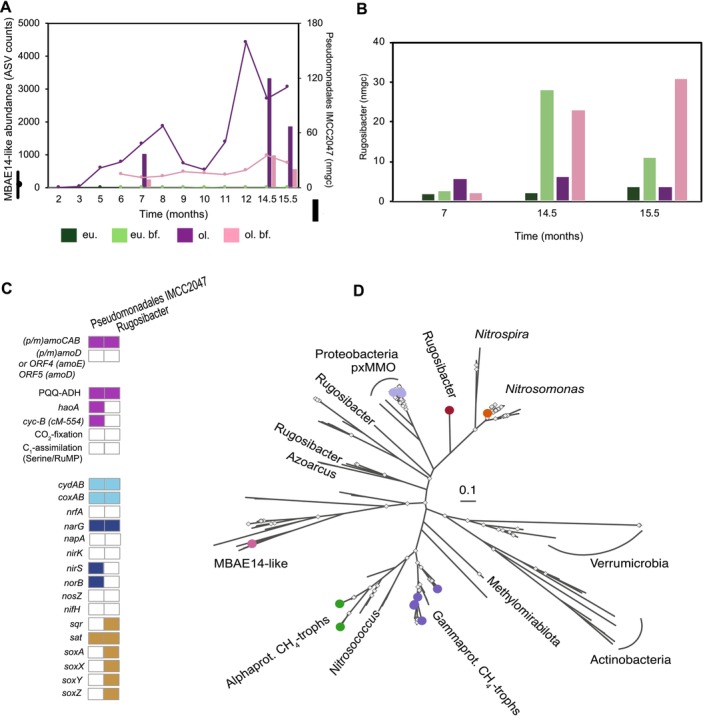
Abundance of novel MBAE14‐like and *Rugosibacter*‐like MAGs, MAG properties and placement of unusual Cu‐MMO sequences in a phylogenetic tree. (A) Primary y‐axis indicates summed MBAE14 family‐like amplicon sequencing variants (ASVs) across time (monthly sampling over the course of the experiment, x‐axis). Secondary y‐axis indicates Pseudomonadales IMCC2047 normalised mean genome coverage (nmgc) on month 7, 14.5 and 15.5 in %. (B) *Rugosibacter* nmgc on month 7, 14.5 and 15.5. (C) Functional annotation of key genes in Pseudomonadales IMCC2047 and *Rugosibacter* MAGs. Question marks indicate either not found in MAG/not present (in canonical form) (D) copper‐dependent monooxygenase (cu‐MMO)‐A protein tree containing binned sequences (colour‐coded) in our study and additional selected Pseudomonadales (in pink) IMCC2047 and *Rugosibacter* (in dark red) GTDB‐Tk genomes containing Cu‐MMO‐A s. For other MAGs the colours refer to current study's binned AOB *Nitrosomonas* (orange), *pxmA*‐containing gammaproteobacterial methanotrophs (light purple), *pmoA*‐containing gammaproteobacterial methanotrophs (dark purple) and alphaproteobacterial methanotrophs (green). Tree scale depicts nucleotide substitutions.

Two high‐quality, novel MAGs associated with anoxic coastal sediments were reconstructed from these metagenomes: the MBAE14‐like gammaproteobacterial Pseudomonadales IMCC2047 and the betaproteobacterial *Rugosibacter* (Figure 5A–C). The Pseudomonadales IMCC2047 genome seemed to be a similar species as the described MBAE14 family‐like Pseudomonadales IMCC2047 (Mori et al. 2019), yet quite different (~ 60% AAI) from the other four Pseudomonadales IMCC2047 genomes available at the GTDB at the time of analysis (Figure S11). The *Rugosibacter* MAG seemed to be quite divergent from the 10 available *Rugosibacter* genomes in the GTDB, sharing only 70% of AAI with the environmental MAG of *Rugosibacter* GCA.002842395.1 (Figure S12).

The MBAE‐14‐like family were only enriched in the oligotrophic bioreactor system from month 5 on, with varying degrees of abundance that peaked during the last months of monitoring (12 to 15.5) (Figure 5A), with values that ranged from 0.04% (oligotrophic bioreactor month 2) to 8.5% (oligotrophic bioreactor month 12) based on the relative bacterial 16S rRNA gene amplicon sequencing results and matching the Pseudomonadales IMMC2047 MAG abundance (Figure 5A). The Pseudomonadales IMCC2047 coverage accounted for between 0.36% (oligotrophic bioreactor biofilm month 7) and 4.5% (oligotrophic bioreactor month 14.5) of the metagenomic reads (Figure 5A and Table S5). The *Rugosibacter* MAG was found from 0.04% (eutrophic bioreactor month 7) to 0.91% (oligotrophic bioreactor biofilm month 15.5) with markedly increasing coverages in the biofilm of both the eutrophic and oligotrophic bioreactor (Figure 5B and Table S5).

The annotations of the Pseudomonadales IMMC2047 and *Rugosibacter* MAGs revealed divergent Cu‐MMOs and PQQ‐dependent alcohol dehydrogenases (ADH) (Figure 5C,D). The recovered MAGs showed that both harboured a complete ‘*p(a)moCAB’* gene‐like cluster, with an additional open reading frame (ORF) that showed low homology to (*p)amoD* (Figure 5C and Table S6). Phylogenetic reconstruction demonstrated that the Cu‐MMO protein sequences from these MAGs were distinct from the homologues encoded by MOB and ammonia monooxygenases (AMOs) of ammonium‐oxidising bacteria (AOB) but harboured all essential amino acids for the coordination of Cu in the active site (Figure 5D and Figure S13). The amino acids of the A subunit of the *Rugosibacter* Cu‐MMO was a lone branch between *amoA* and *pxmA* sequences in a phylogenetic cluster of genome‐derived reference genes (Figure 5D and Figure S13). The gene for the A subunit of the Pseudomonadales IMCC2047 Cu‐MMO was similar to the gene recovered from the Pseudomonadales IMCC2047 (original Mori et al. 2019) and two genes found in MAGs of Gammaprotebacteria (Zhou et al. 2020) (Figure S13). In the Pseudomonadales IMCC2047 MAG, a hydroxylamine oxidoreductase (*haoA*) gene was recovered, without an *haoB* subunit, though it was accompanied by a single cytochrome c‐554 (*cycB*) in the same gene cluster (Figure 5C and Table S6). These genes possibly implicate the Cu‐MMO in this MAG for ammonia rather than methane oxidation; however, many MOBs also encode *haoAB* (Campbell et al. 2011; Versantvoort et al. 2020). Both Pseudomonadales and Rugosibacter MAGs contained a PQQ‐ADH that did not classify with typical calcium (*mxaF*) or lanthanide‐dependent (*xoxF*) methanol dehydrogenases suggesting a different function of the respective proteins (Figure S14). Furthermore, the *Rugosibacter* ADH showed the characteristic conserved domain ‘DXGX(3‐4)D’ essential for the coordination of the lanthanide cofactor, which is also found in *xoxF* sequences (Good et al. 2020).

Contrasting with AOB and MOB, neither of the MAGs encoded the capacity for carbon dioxide assimilation. The complete large and small rubisco subunits required for CO_2_ fixation were missing, and neither of them encoded the Wood‐Ljungdahl pathway nor the reductive tricarboxylic acid (TCA) cycle that AOB utilise to fix CO_2_ (Figure 5C). Moreover, neither of them appeared to be able of canonical methanotrophic C_1_ assimilation via formaldehyde using the serine pathway or the ribulose monophosphate pathway (RuMP). They both lacked a formate‐tetrahydrofolate ligase (FTHFS) encoded by *fhs* (KEGG: K01938) required for the conversion of formate via formyltetrahydrofolate [EC: 6.3.4.3]. Furthermore, we could not find any 3‐hexulose‐6‐phosphate synthase (*hxlA*, KEGG: K08093) [EC: 4.1.2.43] of the ribulose monophosphate (RuMP) pathway (Figure 5C). Ultimately, the lack of any apparent CO_2_ fixation or C_1_ assimilation pathway suggests that these are heterotrophs and thus metabolically distinct from characterised AOB and MOB.

The two MAGs showed potential metabolic flexibility for nitrogen oxide reduction and a certain degree of sulfur cycling. Pseudomonadales IMCC2047 MAG had the potential for partial denitrification, respiring nitrate to nitrous oxide. The *Rugosibacter* MAG encoded nitrate respiration potential together with sulfide detoxification or thiosulfate oxidation (Figure 5C).

## 4 Discussion

Our study investigated the effects of distinct substrate loading regimes of nitrate, sulfide and ammonium on a microbial community under methane saturation in two bioreactors inoculated with sediment from coastal anoxic sediment from the Stockholm Archipelago.

The microbial communities in both systems were quite different from each other after 2 months of operation and remained relatively stable for the remainder of the incubation period (15.5 months; Figure 2A,B and Figure S4). The drop in diversity and richness from eutrophic to oligotrophic systems observed aligned with the general ecological principle of fast‐growing species outcompeting the abundant and diverse rare biosphere. The (gamma)proteobacteria were the most abundant group under all conditions and growth types (bioreactor and biofilm) (Figure 2C and Figure S5). These observations are in line with reports of the uncharacterised metabolic ability of proteobacteria for the utilisation of sulfur and C_1_ compounds in deep‐sea hydrothermal sediments (Zhou et al. 2020). Sulfide detoxification was dominated by nitrate‐reducing sulfide‐oxidising bacteria followed by MOB. In the eutrophic bioreactor, *Campylobacterales* ultimately became enriched over gammaproteobacteria (Figure 2C). Several members of the *Campylobacterales* phylum have been described as opportunistic denitrifiers and seem to be stimulated under low sulfide and high nitrate concentrations (Murphy et al. 2020). In accordance, our two recovered *Campylobacterales* MAGs, *Sulfurimonas* and *Sulfurovum* (Table S5), contained two copies of *sqr* and exhibited either partially complete denitrification potential (*napA*, *nirS*, *norB* and *nosZ*) or only *norB*, respectively (Table S5).

The oligotrophic system sustained DNRA while denitrification dominated in the eutrophic bioreactor (Figure 1A,B). Nitrate limitation as the key factor for DNRA was also observed in other studies (Arshad et al. 2017; Dalcin Martins et al. 2022; Berg et al. 2016). Although previous studies reported inhibition of denitrification and DNRA by sulfide (Delgado Vela et al. 2021; Russ et al. 2014; Zhang et al. 2024), in our systems we did not observe this inhibition, possibly because the sulfide was directly consumed by the microbial community and thus the in situ sulfide concentration remained below the detection limit. Similar observations have been made for a sulfide and methane‐oxidising co‐culture where a new *Nitrobium* species was enriched (Arshad et al. 2017; Dalcin Martins et al. 2022). Another study also found that sulfur disproportionation could fuel DNRA, a reaction that might have occurred in our oligotrophic system (Shao et al. 2024).

Aerobic methanotrophs of the *Methylomonadaceae* type I (gammaproteobacteria) constituted a significant portion of the total bacterial reads in both systems, despite the supply of sulfide and nitrate (Figure 3A, Figure S3 and Tables S1 and S2). Abundant persistent populations of gammaproteobacterial methanotrophs have been described in oxygen‐depleted lakes and wetlands and marine oxygen minimum zones (Oswald et al. 2016; Schorn et al. 2024; Smith et al. 2018; Smith and Wrighton 2019). In this regard, some of the physiological and genomic adaptations for survival under oxygen‐limited conditions of MOB have been speculated to include denitrification potential, fermentation pathways, high‐affinity oxidases, oxygen carriers like bacteriohemrythrins, and gas vesicles (Reis et al. 2024).

When further exploring the methanotrophic community of our two systems, the species belonging to the family *Methylomonadaceae* showed the potential for sulfide detoxification and partial denitrification (Figure 4 and Figure S8). The relevance of these gammaproteobacterial MOBs in C, N and S cycling has been also documented in freshwater canal biofilms, lake sediments, and hypoxic eutrophic coastal system (Deng et al. 2024; Pelsma et al. 2023; Venetz et al. 2023). Members of the genus *Methylomonas* seem to thrive very well under oxygen limitation as was first documented in 2015 (Kits, Klotz, and Stein 2015), and later confirmed in other systems such as nitrate‐reducing methane‐oxidising anoxic bioreactors systems (Guerrero Cruz et al. 2018). In addition to the use of oxidised nitrogen compounds, these *Methylomonas* seem well‐equipped to deal with reduced S compound oxidation and have the potential to disproportionate thiosulfate (S_2_O_3_^2−^), tetrathionate (S_4_O_6_^2−^), elemental sulfur (S^0^) or sulfide, as was likewise described in the pure culture of the alphaproteobacterial *Mehylovirgula thiovorans* HYI and Verrucomicrobial methanotroph *Methylacidiphilum fumariolicum* SoIV (Gwak et al. 2022; Schmitz et al. 2023). Both the genus *Methylovulum* and *Methylomonadaceae* subgroup KS41 showed preferential enrichment under lower sulfide loading in the biofilm (Figure 4A, Figure S9 and Table S3). While for the genus *Methylovulum*, there are cultured representatives (Iguchi, Yurimoto, and Sakai 2011; Oshkin et al. 2016) that could be tested, the genus *Methylomonadaceae* KS41, with the highest amount of reads, has no cultured representatives yet (Figure S15 and Table S7). Better resolving the activities and interaction of C, N and S pathways in methanotrophs may have substantial implications for understanding and modelling biogeochemical cycling in these ecosystems.

Freshwater ‘*Ca*. Methanoperedens BLZ2’ *sp*. was the only archaeal methanotroph that became enriched from 7 months onwards in the oligotrophic system, reaching as much as 21% of the total metagenomic reads in month 15.5 (Figures S5 and S7 and Table S5) despite the 2% brackish salinity in the medium. ‘*Ca*. Methanoperedens BLZ2’ showed a considerable contribution to DNRA via *nrfA* under nitrate‐limiting and methane‐saturated conditions, reaching more than 80% of *nrfA* reads mapping to ‘*Ca*. Methanoperedens BLZ2’ at month 15.5, similar to what was found in previous studies (Arshad et al. 2017; Dalcin Martins et al. 2022) (Figure 3B and Figures S5 and S7). The observed co‐occurrence of ‘*Ca*. Methanoperedens’ spp. with *Methylomonadaceae* type I methanotrophs was also described in a hypoxic marine eutrophic lake, indicating their potential prevalence under nitrate‐limiting conditions (Venetz et al. 2023).

Together with abundant MOBs, ‘*Ca*. Methanoperedens BLZ2’ *sp*. and sulfur oxidisers, we co‐enriched novel bacteria affiliated with Pseudomonadales IMCC2047 and *Rugosibacter* (Figure 5). Both corresponding MAGs contained unusual Cu‐MMO‐A sequences that clustered separately in an extended Cu‐MMO tree (Figure 5D and Figure S13). Both MAGs also included a complete *(p/a)moCAB*‐like gene operon, followed downstream by a single putative *amoD/pmoD*‐like ORF (Koo and Rosenzweig 2020) (Figure 5C and Table S6). There is no consensus in literature for describing canonical *amoCAB‐*associated *ED* subunits, due to a variation in the protein family level annotation. Another shared feature of the Pseudomonadales IMCC2047 and *Rugosibacter* MAGs is the lack of known CO_2_ fixation or formaldehyde‐derived C_1_‐assimilation pathways (serine and RuMP) characteristic for AOBs or MOBs (Kalyuzhnaya, Gomez, and Murrell 2019), and therefore are apparent heterotrophs with unknown function(s) of their Cu‐MMO. A third common shared unusual observation in these two genomes is the presence of PQQ‐dependent ADHs (Figure 5C, Figure S14 and Table S6). MOBs and methylotrophs employ PQQ‐ADH‐like lanthanide (*xoxF*) or calcium‐dependent (*mxaF*) methanol dehydrogenases and related ADHs to oxidise alcohols to aldehydes, a genetic repertoire that AOBs do not share (Kikuchi et al. 2023; Kuypers, Marchant, and Kartal 2018). Neither of the new PQQ‐ADHs clustered near methanol‐dependent dehydrogenases (Figure S14) but seemed to be more affiliated with alcohol/ethanol dehydrogenases (Keltjens et al. 2014). Therefore, rather than methanol, the PQQ‐dependent‐ADH may be oxidising short‐chain alcohols that are possibly fermentation products of other community members.

One clear genetic feature that distinguished Pseudomonadales IMCC2047 from *Rugosibacter* was the presence of a hydroxylamine oxidoreductase *haoA* with a cytochrome c‐544 (*cycB*) encoded in the same operon (Table S6). It is known that MOB can use Hao to detoxify hydroxylamine under high ammonium loads (Poret‐Peterson et al. 2008). In contrast, AOB can conserve energy from hydroxylamine oxidation by employing two cytochromes in the same operon (*haoAB*, *cycAB*), funnelling electrons to the quinone pool and generating a proton motive force. Some exceptions to the classic AOB operon organisation include ‘*Ca*. Nitrosacidococcus tergens’ sp. RJ19, which has a *haoAB‐cycB* structure (Picone et al. 2021). Additionally, quite often AOB genomes encode multiple copies of hydroxylamine oxidoreductase genes compared to MOB (Kikuchi et al. 2023). Moreover, Pseudomondales IMCC2047 lacks the *haoB* subunit, the signature catalytic subunit of Hao in AOBs (Kikuchi et al. 2023). Still, some MOBs like *M. capsulatus* Bath also harbour Hao homologues *haoA* and *haoB* subunits together (Poret‐Peterson et al. 2008). These contrasting AOB and MOB genetic signatures all together highlight the atypical nature of Pseudomonadales IMCC2047 *(p/a)moCAB* gene operon‐like and *haoA*‐*cycB* operon structures and metabolic capabilities.

The first MBAE14/Pseudomonadales IMCC2047 MAG was described in 2019 in a similar ecosystem with oxygen limitation and the presence of sulfide, methane, and ammonium (Mori et al. 2019). Based on the genomic potential, it was postulated to act as a putative mixotrophic nitrifier‐denitrifier. The MBAE14 MAG reconstructed here seems unlikely to be a functional nitrifier; instead, the substrate range of the unusual Cu‐MMOs should be further investigated (e.g., by (meta)transcriptomics) as it could provide key insights into the ecological niche of this bacterium. Another insight from Mori et al. and our study is that the MAG has high AAI similarity to marine heterotrophs, including *Oleiphilus* spp. (> 90%), *Marinobacter* spp. (> 90%) and the denitrifier *Pseudomonas stutzeri* (> 88.4%), and heterotrophic nitrifier/denitrifier *Alcaligenes faecalis* (> 78.9%) (Mori et al. 2019). The microorganisms are known to oxidise aliphatic (*Oleiphilus* spp. and *Marinobacter* spp) or aromatic ( *Pseudomonas stutzeri* ) hydrocarbons (Bowman and McMeekin 2015; Lenferink et al. 2024; Singh and Tiwary 2017; Yakimov, Timmis, and Golyshin 2007). *Alcaligenes faecalis* can produce large amounts of hydroxylamine during heterotrophic nitrification (Lenferink et al. 2024). When building the Cu‐MMO tree, we also included sequences belonging to the *Azoarcus* genus (Figure 5D), as both genomes from the genus *Pseudomonas* and *Azoarcus* have been co‐enriched in a membrane‐aerated nitrification–denitrification tank (Lan et al. 2023).

Lastly, we describe a novel *Rugosibacter* MAG (Figure S13) that was present in the biofilms of both eutrophic and oligotrophic bioreactors (0.91% enriched at month 15.5) (Figure 5B and Table S3). This genus has only a single cultured isolate, which lacks a Cu‐MMO but can degrade aromatic compounds (Corteselli, Aitken, and Singleton 2017). Interestingly, the PQQ‐ADH of this MAG shows a high similarity (100% query coverage, 94.72% identity) to the one of an unpublished groundwater MAG of *Rugosibacter*, with cryptic sulfur and nitrogen cycling potential (Locus: MDO8347641; Bioproject: PRJNA700657). However, this putative groundwater *Rugosibacter* MAG lacks a Cu‐MMO, which is often mis‐binned and may be missing from the published *Rugosibacter* MAG.

Salinity was increased from 1% to 2% over the course of 1 month, from months 14.5 to 15.5 (Figure S1). We observed no salinity effect on the microbial community composition (Figure 2, Figure S5 and Tables S1–S3) or functional potential (Figure 1, Figure S5 and Table S5), which remained stable. The salinity increase tolerance observed for the freshwater ‘*Ca*. Methanoperedens BLZ2’ sp. (Figure S15) aligned with the acclimatation described in studies on brackish‐acclimated (1.5%) cultures of ‘*Ca*. Methanoperedens nitroreducens’ sp. (up to 3.7% salt increase) (Frank et al. 2023, Echeveste Medrano et al. 2024). Likewise, the *Methylomonadaceae* family also included marine representatives (Figure S16), suggesting potential for marine adaptation.

To conclude, in this investigation, we studied the effect of distinct nitrogen and sulfide additions on two coastal anoxic sediment microbiomes. We concluded that Gammaproteobacteria are the most dominant and distinct group emphasising their ability to deal with sulfide stress and that nitrate limitation stimulated DNRA that can accept more electrons per nitrate molecule compared to denitrification. The poorly characterised *Methylomonadaceae* genus *Methylovulum* and subgroup KS41 showed high metabolic flexibility and appeared to be strong competitors for nitrate reduction with the anaerobic methane oxidiser ‘*Ca*. Methanoperedens BLZ2’ spp. Furthermore, we retrieved MAGs of Pseudomonadales IMCC2047 and *Rugosibacter* with very unusual Cu‐MMOs whose functions need to be further evaluated.

## Author Contributions

**Maider J. Echeveste Medrano:** conceptualization, investigation, methodology, formal analysis, writing – original draft, writing – review and editing, software. **Garrett J. Smith:** methodology, software, formal analysis, writing – review and editing. **Irene Sánchez‐Andrea:** funding acquisition, writing – review and editing, supervision. **Mike S. M. Jetten:** supervision, writing – review and editing, funding acquisition, conceptualization. **Cornelia U. Welte:** funding acquisition, writing – review and editing, supervision, conceptualization.

## Conflicts of Interest

The authors declare no conflicts of interest.

## Supporting information


Appendix S1.

Appendix S2.

## Data Availability

Raw 16S rRNA gene sequences and metagenomic reads and genomes have been uploaded to the European Nucleotide Archive (ENA) with project number PRJEB79860. Supporting Information Tables are available on zenodo under doi:10.5281/zenodo.14004133 (https://zenodo.org/records/14004133).
