# Supplementary material for: Contrasting Methane, Sulfide and Nitrogen‐Loading Regimes in Bioreactors Shape Microbial Communities Originating From Methane‐Rich Coastal Sediment of the Stockholm Archipelago
Source: Environ Microbiol. 2025 Feb 16;27(2):e70056. doi: 10.1111/1462-2920.70056 (PMC11830464; doi:10.1111/1462-2920.70056)
Supplement: Supplementary file 1 — Appendix S1. [file EMI-27-e70056-s002.docx]

**Appendix S1**

**Supplemental Tables 1-7 as a single excel file** **are available on zenodo:** **https://zenodo.org/records/14004133**



**Supplementary Figure 1.** Inoculation and sampling workflow. On the left, the sediment core depth-mix used for inoculation (9-16cm) is indicated in white on the sediment core. Anoxic inoc. mix (anoxic inoculum mix), eutro (eutrophic), oligo (oligotrophic), br (bioreactor), bf (biofilm).

**Supplementary Figure 2.** Eu(trophic) (left) and oligo(trophic) (right) bioreactor images obtained at month 5, when biofilm growth became apparent in both bioreactors. Biofilm (bf.) was only collected for DNA extractions from month 6 onwards.


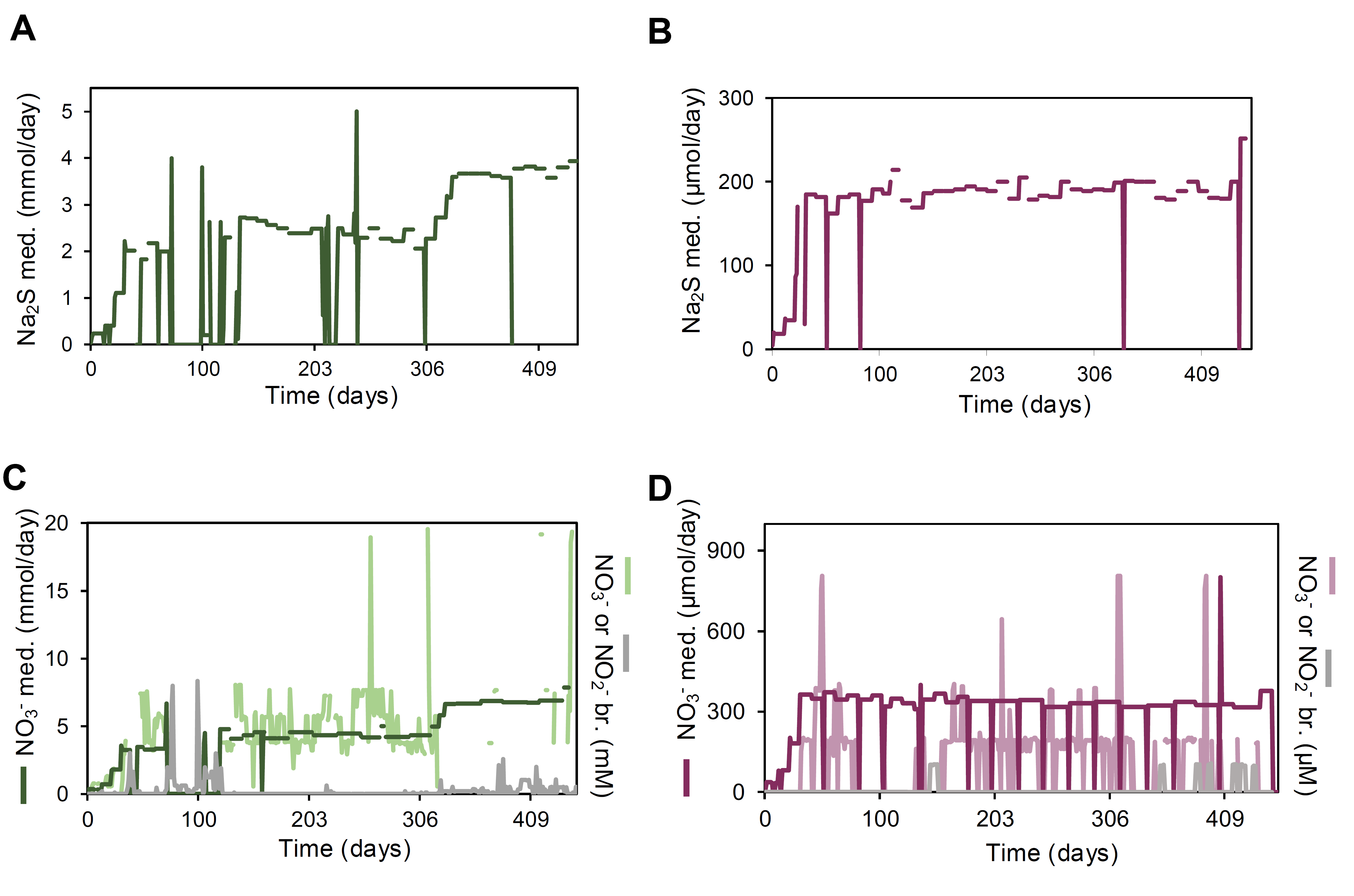


**Supplementary Figure 3. A-B** Absolute amount of sulfide (Na_2_S x 3 H_2_O) added to the eutrophic (A) and oligotrophic (B) bioreactors over the course of the experiment (x-axis in days). Sulfide was completely metabolized by the microbial community and not detected above the detection limit (picomolar) during the experiment inside the reactor vessel for both systems **C-D** Absolute amounts of nitrate added to the eutrophic (C) and oligotrophic (D) bioreactors in mmol per day on the primary y-axis; the secondary y-axis shows the daily measuredy nitrate (NO_3_^-^) and nitrite (NO_2_^-^) concentrations measured in the bioreactors over the course of the experiment (x-axis in days). Both primary and secondary y-axes indicate the same scale but with different units. Note that the eutrophic and oligotrophic substrate bioreactor concentrations are indicated in mmol and µmol, respectively.


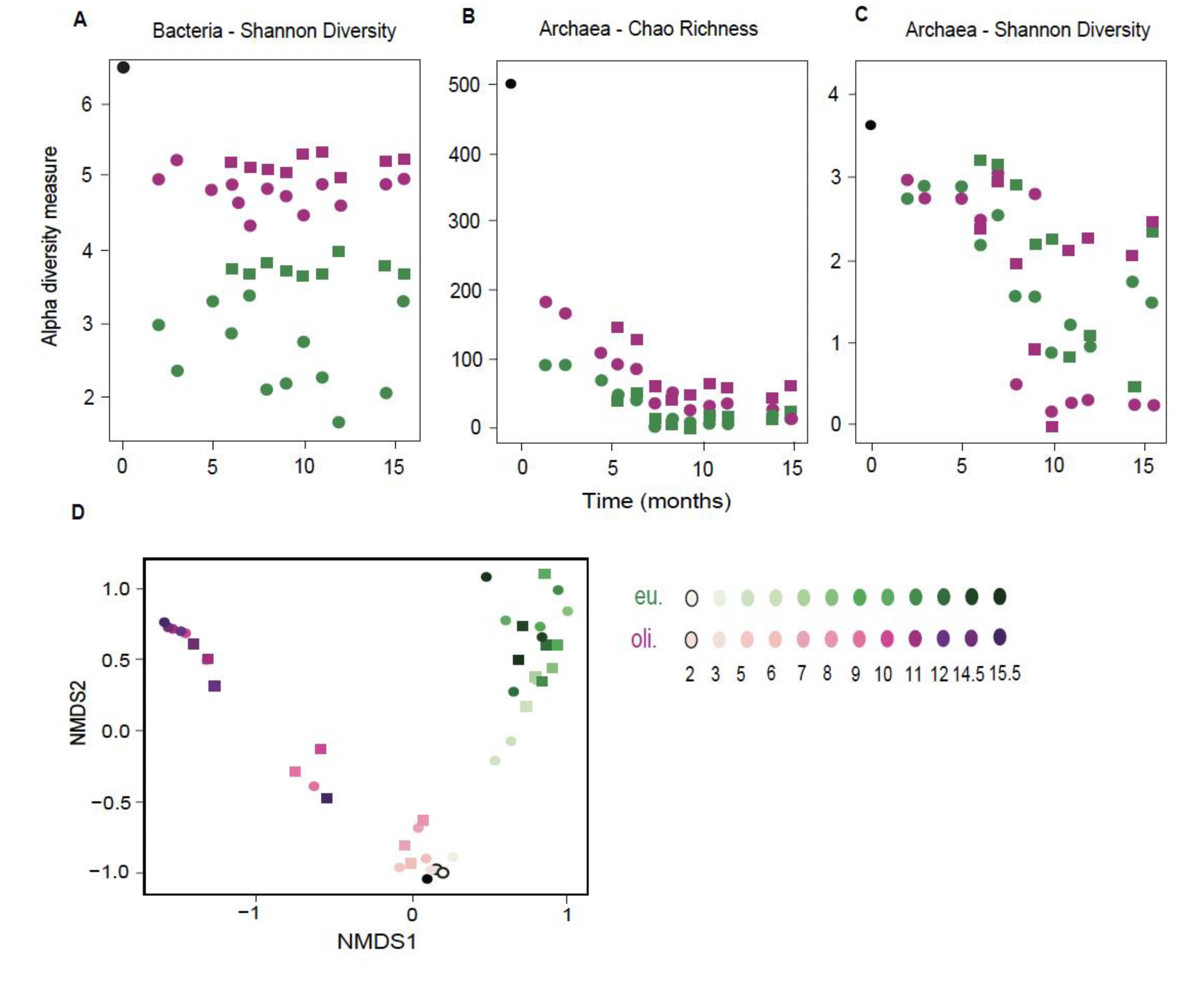


**Supplementary Figure 4. A** Bacterial 16S rRNA amplicon sequencing variants (ASVs) diversity index was calculated via Shannon index and indicated as Alpha diversity measure (y-axis) per sample source across time (x-axis). Black circle, inoculum. **B-C** Archaeal 16S rRNA amplicon ASVs richness (B) and diversity (C) calculated using Chao or Shannon indices, respectively. Eutrophic (eu.) and oligotrophic (ol.) system sampling was color-coded in green and pink, respectively. Shapes indicate inoculum (inoc.), bioreactor (br.) and biofilm (bf.) in filled circle, green or pink colored circle or green or pink square, respectively. Black circle, inoculum. **D** Nonmetric multidimensional scaling (NMDS) based on Bray-Curtis Dissimilarity of 16S rRNA archaeal ASVs per bioreactor substrate loading. Dimensionality was set at k=4 and recorded a stress value of 0.04831986.

**Supplementary Figure 5. A-B** Z-score mean normalized genome coverage of highly curated genomes (>90% complete and <5% contaminated) with > 1% of mapped reads for any time point, excluding the inoculum sediment depths. Panel A and B indicate the abundance of specific genomes with the eutrophic (A) or the oligotrophic bioreactor as query.. MAGs were classified to the lowest known GTBD-Tk taxonomical category. Columns within sample groups represent the three sampled time points (month 7, 14.5 and 15.5).

**
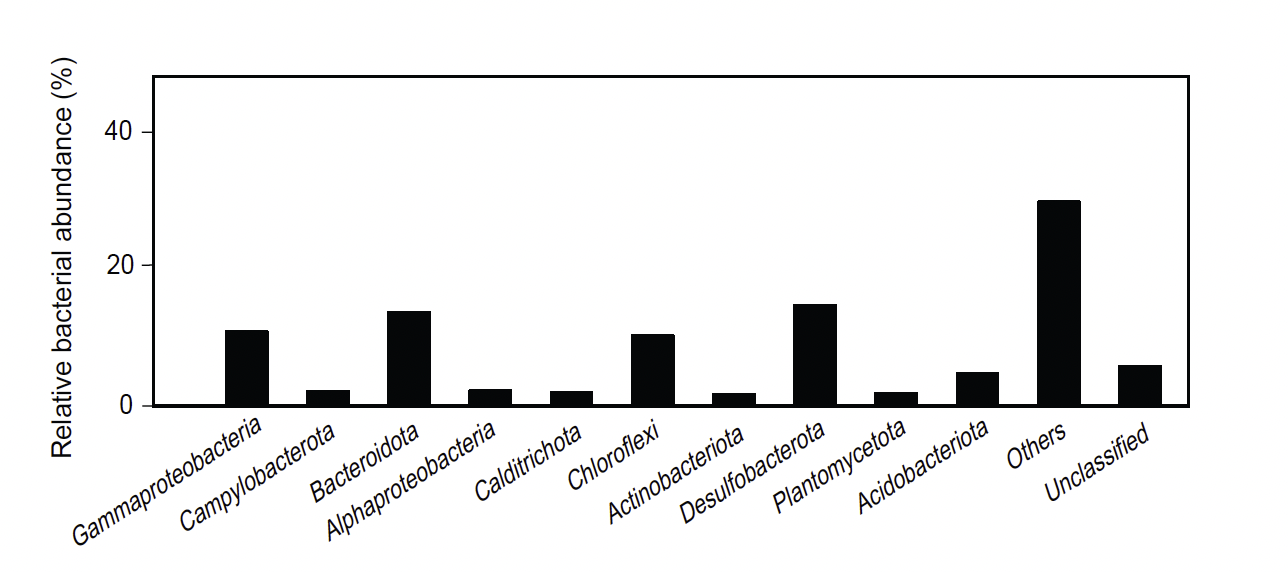
Supplementary Figure 6.** Bacterial 16S rRNA gene Amplicon Sequencing Variants (ASVs) reads (>5% abundant) recovered at phylum level for the inoculum.

**Supplementary Figure 7.** Total ‘*Ca.* Methanoperedens’ Amplicon Sequencing Variants (ASVs) recovered across time and conditions represented as stacked mountain plot: eu(trophic) or, ol(igotrophic) and with/without biofilm (bf.).

**Supplementary Figure 8.** Percentage of top (> 10%) methanotrophic genome contribution to functional marker gene coverage for nitrate assimilation (*nirB*) and denitrification (*narG, napA, nirS, nirK, norB, nosZ*).

**Supplementary Figure 9.** GTBD-Tk genome tree of recovered *Methylomonadaceae* Metagenome Assembled Genomes (MAGs) (in orange). *Methylomonadaceae* MAGs of interest were annotated either in green or pink if they were more abundant in either in the high or low system, respectively (Figure 4A). Different blue shades depict different genera. Tree scale depicts amino acid substitutions.

**
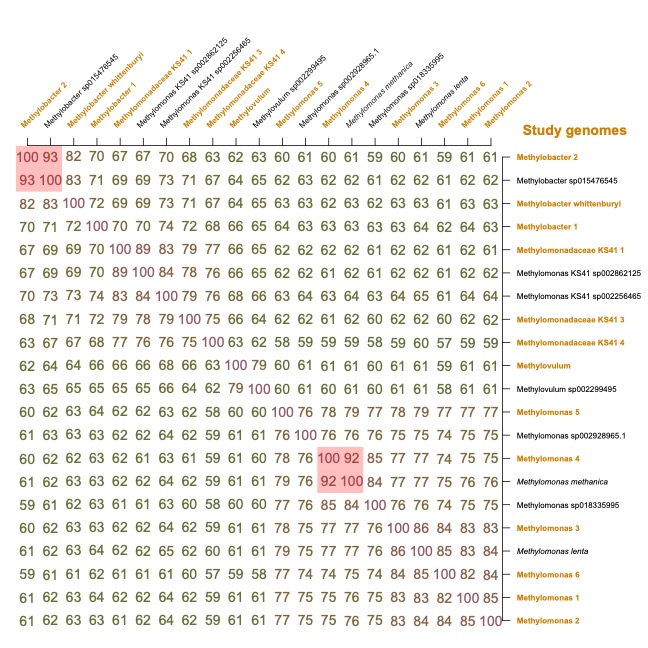
**

**Supplementary Figure 10.** Fast Average Amino acid (AAI) identity matrix of recovered *Methylomonadaceae* Metagenome Assembled Genomes (MAGs) (in orange). Reference isolated or environmental publicly available MAGs were selected based on proximity genome tree (Supplementary Figure 5). Values ≥ 90% are highlighted in red to indicate same species based on (Rodriguez-R & Konstantinidis, 2016).

**
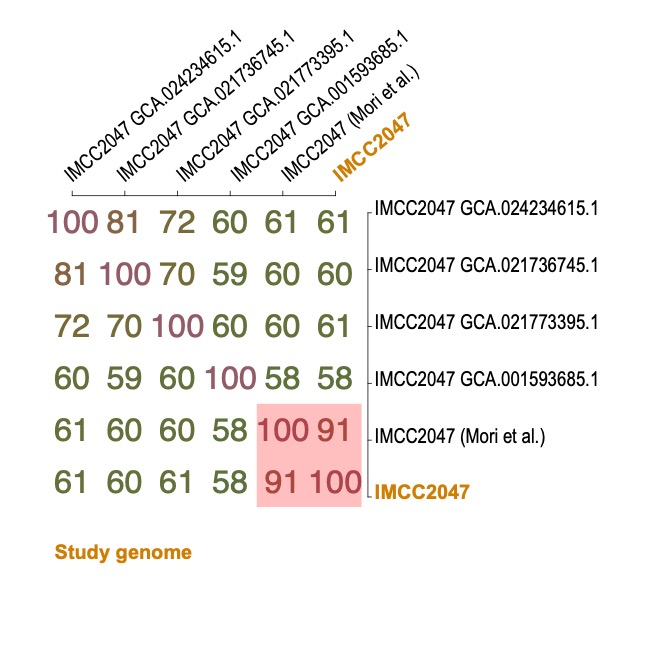
**

**Supplementary Figure 11.** Fast Average Amino acid (AAI) identity matrix of our study’s Pseudomonadales IMCC2047 genus Metagenome Assembled Genomes (MAG) (in orange) against reference GTDB genomes plus Mori et al 2019 Pseudomonadales IMCC2047 genome. Values ≥ 90% are highlighted in red to indicate same species based on (Rodriguez-R & Konstantinidis, 2016).


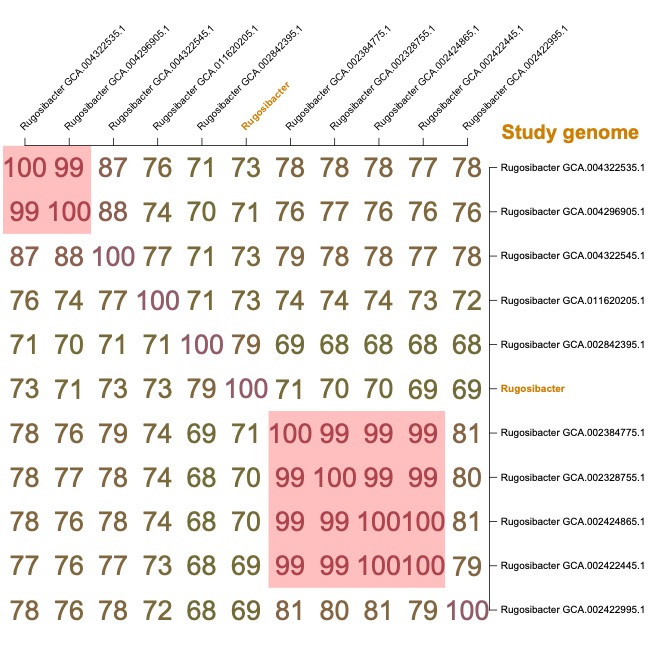


**Supplementary Figure 12.** Fast Average Amino acid (AAI) identity matrix of our study’s *Rugosibacter* Metagenome Assembled Genomes (MAG) (in orange) against reference GTDB genomes. Values >90% are indicated in red to indicate same species. Values ≥ 90% are highlighted in red to indicate same species based on (Rodriguez-R & Konstantinidis, 2016).

**
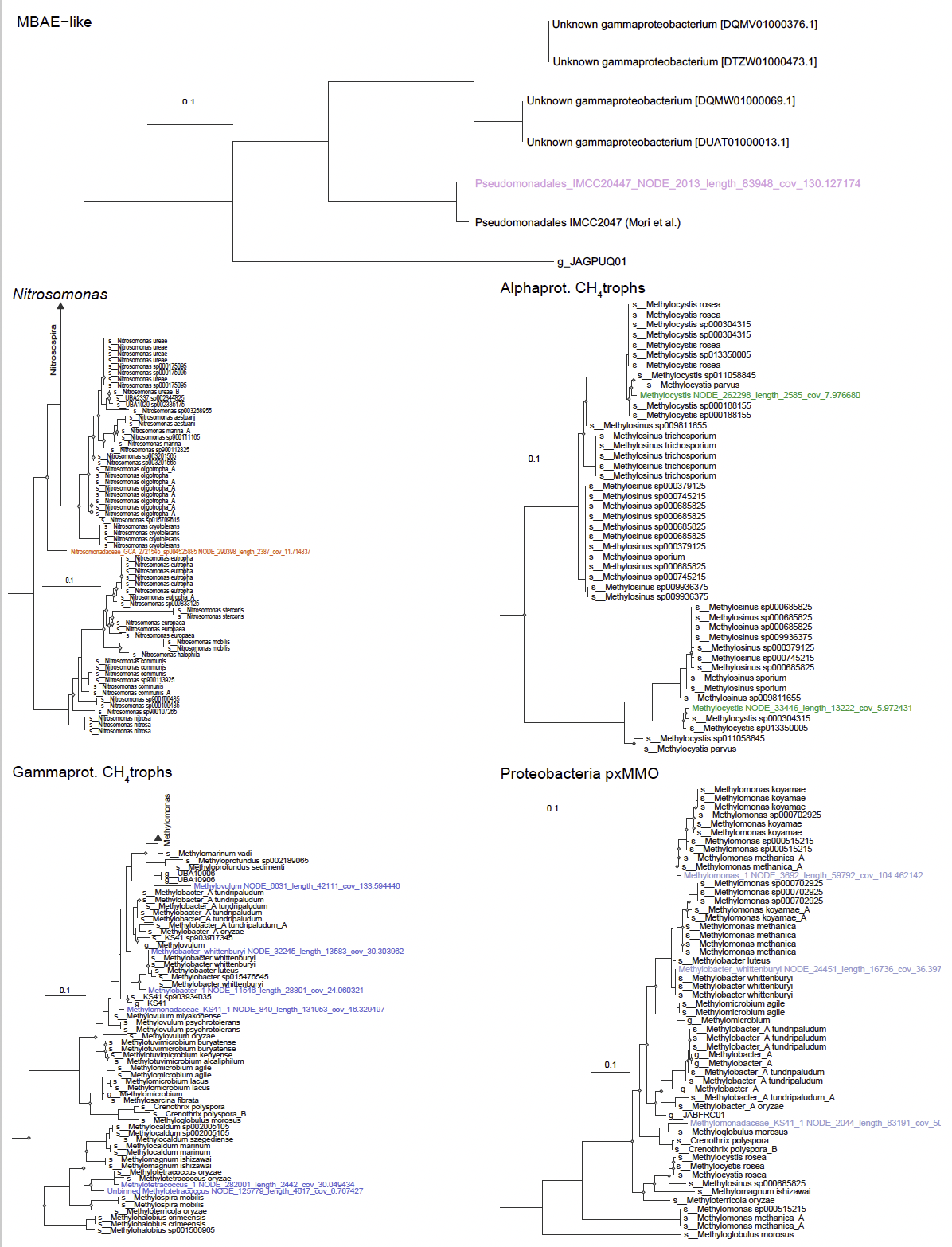
**

**Supplementary Figure 13.** Zoomed in version of selected microbial groups containing Copper-dependent monooxygenase (Cu-MMO)-A that got binned (and color-coded) on a Metagenome Assembled Genome (MAG) in the present study. Color coding aligns with the one present on the unrooted phylogeny of Figure 5D. Colors refer to binned MBAE14-like family (pink), AOB *Nitrosomonas* (orange), alphaproteobacterial methanotrophs (green), *pmoA*-containing gammaproteobacterial methanotrophs (dark purple) and *pxmA*-containing gammaproteobacterial methanotrophs (light purple). Tree scale depicts amino acid substitutions.

**Supplementary Figure 14.** Pyrroloquinoline quinone (PQQ)-dependent alcohol dehydrogenases (DHs) tree with selected genomes harboring either unknown alcohol dehydrogenases, lanthanide-dependent methanol dehydrogenases (*xoxF*) and calcium-dependent methanol dehydrogenases (*mxaF*) in pink, green and gold, respectively. Our study genomes and reference MBAE14-genome (Mori et al 2019) with unknown PQQ-dependent dehydrogenases are labelled in orange or light blue, respectively. Sequences were aligned using MAFFT, trimmed (trimal –gappy out), ran in IQtree with the Maximum Likelihood method with a minimum of 1000 bootstraps replicates. Final annotations were done using iTOL. Tree scale depicts nucleotide substitutions.

**Supplementary Figure 15.** SingleM-based Sandpiper results of species ‘*Ca.* Methanopereredens BLZ2’ (GCA_002487355.1) in the Sequencing Read Archive (SRA) (total of 52). Plotted pie chart indicates metagenome sample source (biosample) counts per different engineered/natural ecosystems. “Others” category compiles metagenomes with biosample source indicated as “metagenome”.

**Supplementary Figure 16.** SingleM-based Sandpiper results of our study’s main *Methylomonadaceae* genus: KS41, *Methylomonas*, *Methylovulum* and *Methylobacter* (x-axis). Y-axis indicates counts per sequencing reads archive (SRA) metagenomic project and are classified from highest to lowest counts per different top ecosystems. “Others” category compiles metagenomes with biosample labels that accounted for ecosystems with representation below the top 10 of SRA counts for each genus. Genus KS41 showed the highest SRA hits with compared to *Methylobacter* (ordered from highest to lowest count in the x-axis).
